# Membrane-Type 5 Matrix Metalloproteinase (MT5-MMP): Background and Proposed Roles in Normal Physiology and Disease

**DOI:** 10.3390/biom15081114

**Published:** 2025-08-03

**Authors:** Deepak Jadhav, Anna M. Knapinska, Hongjie Wang, Gregg B. Fields

**Affiliations:** Institute for Human Health & Disease Intervention (I-HEALTH) and Department of Chemistry & Biochemistry, Florida Atlantic University, Jupiter, FL 33458, USA; djadhav@fau.edu (D.J.); aknapins@fau.edu (A.M.K.); wangh@fau.edu (H.W.)

**Keywords:** membrane-type matrix metalloproteinase, MT5-MMP, Alzheimer’s disease, cancer

## Abstract

The matrix metalloproteinase (MMP) family includes several membrane-bound enzymes. Membrane-type 5 matrix metalloproteinase (MT5-MMP) is unique amongst the MMP family in being primarily expressed in the brain and during development. It is proposed to contribute to synaptic plasticity and is implicated in several pathologies, including multiple cancers and Alzheimer’s disease. In cancer, MT5-MMP expression has been correlated to cancer progression, but a distinct mechanistic role has yet to be uncovered. In Alzheimer’s disease, MT5-MMP exhibits pro-amyloidogenic activity, functioning as an η-secretase that cleaves amyloid precursor protein (APP), ultimately generating two synaptotoxic fragments, Aη-α and Aη-β. Several intracellular binding partners for MT5-MMP have been identified, and of these, N4BP2L1, EIG121, BIN1, or TMX3 binding to MT5-MMP results in a significant increase in MT5-MMP η-secretase activity. Beyond direct effects on APP, MT5-MMP may also facilitate APP trafficking to endosomal/lysosomal compartments and enhance proinflammatory responses. Overall, the substrate profile of MT5-MMP has not been well defined, and selective inhibitors of MT5-MMP have not been described. These advances will be needed for further consideration of MT5-MMP as a therapeutic target in Alzheimer’s disease and other pathologies.

## 1. Background

**Figure 1:**
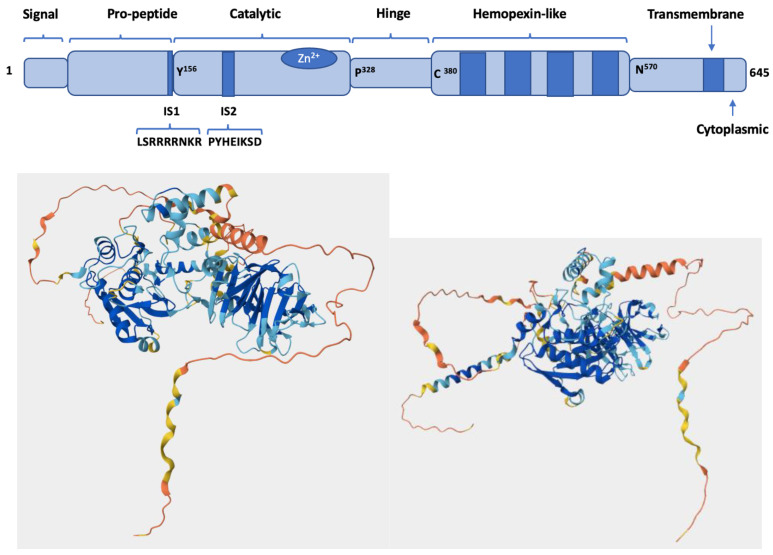
Structure of MT5-MMP indicated by (**top**) domain organization [1] and (**bottom left and right**) three-dimensional fold (AlphaFold; https://alphafold.ebi.ac.uk/entry/Q9Y5R2 (accessed on 16 May 2025)). The predicted structure for MT5-MMP (3 alpha-helices and 5 beta-sheets within the globular catalytic domain, left blue-colored domain in bottom-left figure; 4 bladed propeller hemopexin-like domain with each blade containing 4 antiparallel beta-strands, right blue-colored domain in bottom-left figure) is analogous to other MMP family members. For the AlphaFold structure, dark blue is very high confidence (per-residue confidence score (Local Distance Difference Test; pLDDT) > 90), light blue is moderate confidence (90 > pLDDT > 70), yellow is low confidence (70 > pLDDT > 50), and orange is very low confidence (pLDDT < 50).

Membrane-type 5 matrix metalloproteinase (MT5-MMP/MMP-24) contains 645 amino acids with a predicted molecular mass of 73.2 kDa [1]. As a member of the MT-MMP family, it shares a common domain structure consisting of a signal peptide, a pro-domain, a catalytic domain, a linker, a hemopexin-like domain, a stalk region, a transmembrane domain, and a short cytoplasmic domain (Figure 1) [2,3]. The overall folds of the catalytic and hemopexin-like domains are predicted to be similar to other MMP family members (Figure 1). The zinc-containing catalytic domain catalyzes peptide bond hydrolysis, while the hemopexin-like domain facilitates the binding of macromolecular substrates. Several mechanisms by which MMPs catalyze peptide hydrolysis have been proposed [4,5,6,7]. The linker typically plays a key role in MMP processing of macromolecular substrates, as the favorable positioning of the catalytic domain in proximity to the hemopexin-like domain is dependent on the flexibility of the linker [8,9]. While the role of the MT5-MMP cytoplasmic domain is relatively unexplored, the MT1-MMP cytoplasmic domain has been shown to modulate cellular activities via posttranslational modifications of the domain [10,11].

Initially reported in the brain, kidney, pancreas, and lung and during embryonic development [1,12,13], its expression is most prominent in the neuronal cells of both central and peripheral nervous systems and in mast cells [14,15,16,17,18,19]. It is also expressed in calcitonin-gene-related peptide-positive (CGRP^+^) dorsal root ganglion neurons [14,18] and is elevated in the adult hippocampus, olfactory bulb, and cerebellum [20].

ProMT5-MMP has been shown to be activated by furin in neurons [17] and by propeptide convertase subtilisin/kexin type 6 (PCSK6) in mouse neuroblastoma (N2A) cells stably expressing human Swedish mutant APP695 (N2A^APP^) [21]. PCSK6 recognizes the Arg-Arg-Arg-Asn-Lys-Arg sequence within the propeptide [21], and furin-mediated activation occurs in the trans-Golgi network [22].

Unlike MT1-MMP, MT5-MMP internalization is independent of the dynamin-regulated endocytic pathway [23]. Once internalized, it colocalizes with the early endosomal marker RUN and FYVE domain containing 1 (RUFY1), indicating delivery to the early endosomes after internalization [23]. Mint-3 regulates the retrieval of internalized MT5-MMP to the plasma membrane by binding to its carboxyl end motif Glu-Trp-Val [23]. Both the phosphotyrosine-binding (PTB) and PDZ domains of Mint-3 contribute to this regulation, which occurs in the trans-Golgi network [23]. Interestingly, while low levels of Mint-3 support MT5-MMP return to the cell surface, higher Mint-3 levels inhibit it in both HEK293 cells and Neuro2A neuronal cells—an effect that appears to be non-specific [23]. MT5-MMP is active at the cell surface and intracellularly [12,24]. MT5-MMP was also found to be shed in active form from Madin–Darby canine kidney (MDCK) cells and human breast cancer MCF-7 and T47D cells [12].

MT5-MMP binds α-amino-3-hydroxy-5-methyl-4-isoxazolepropionic acid (AMPA) receptor binding protein (ABP) and glutamate receptor interacting protein (GRIP), where those interactions may localize MT5-MMP and contribute to synaptic remodeling [17]. MT5-MMP was originally identified from rat brain when screening for the interacting proteins of the *C*-terminus of ABPs (amino acids 295–822) [17,23,25].

In the rat, expression of MT5-MMP is first detected in the embryonic-day-16 brain, increases at embryonic day 20, and peaks at postnatal day 0 throughout the brain parenchyma [20]. Postnatal expression decreases to adult levels by day 60 [20]. During development the enzyme expression is detected in the central nervous system and the peripheral nervous system, including the trigeminal ganglion and dorsal root ganglia [20], and has also been detected in the thymus and aorta [20]. MT5-MMP has been proposed to contribute to synaptic plasticity [20] and to the development of dermal neuro-immune synapses [18].

MT5-MMP participates in axodendritic development and remodeling after nerve injury [14,15,19] and is essential for neuronal cell migration and neurite formation [14,26]. It is found in axonal growth cones and developing dendritic spines [14]. In its absence, neurons exhibit increased spontaneous depolarizations and are resistant to interleukin-1β (IL-1β)-induced hyperexcitability [27].

Neural stem cell quiescence is due to N-cadherin-mediated anchorage to ependymocytes [28]. MT5-MMP sheds the N-cadherin ectodomain and is necessary for the activation of B-cells [28]. Elevated levels of the enzyme were found to colocalize with N-cadherin in glial fibrillary acidic protein-positive (GFAP^+^) B1 and ependymal cells [28].

## 2. MT5-MMP Substrates

MT5-MMP was initially described as an activator of proMMP-2 [1,12] in a tissue inhibitor of metalloproteinase 2 (TIMP-2)-dependent manner, in similar fashion to MT1-MMP [29]. It was not found to activate proMMP-9 [1].

Known substrates include gelatin [12,29], laminin-1 [29], fibronectin [29], N-cadherin (CDH2) [17], E-cadherin (CDH1) [17], KiSS-1 [25], the KiSS-1-derived decapeptide metastin [25], Nogo-66 receptor 1 (NgR1, RTN4R) [30], amyloid precursor protein (APP) [31,32], crystallin αB [33], and myelin basic protein (MBP) [34]. The data indicated partial cleavage of laminin, although it was reported that laminin was resistant to MT5-MMP digestion [29]. MT5-MMP can digest MBP, but is the least efficient when compared to other MT-MMPs [34]. The enzyme degrades itself rapidly at 37 °C [29].

MT5-MMP cleaves chondroitin sulfate proteoglycans (CSPGs) and dermatan sulfate proteoglycans (DSPGs) well and fibronectin more slowly [29]. Ectodomain cleavage of N-cadherin generates a ~35 kDa membrane-bound *C*-terminal fragment (CTF1), which is further cleaved by γ-secretase or the proteasome [17,28]. MT5-MMP mediates peripheral thermal nociception and inflammatory hyperalgesia by shedding N-cadherin [18]. Although MT5-MMP was not specifically identified, cleavage of E-cadherin is carried out by a membrane-bound metalloproteinase [35], occurring at Pro^700^-Val^701^, which produces a 38 kDa membrane-bound fragment [36]. The enzyme has been reported to bind to CD44 [37] but it does not cleave it [38,39].

**Figure 2:**
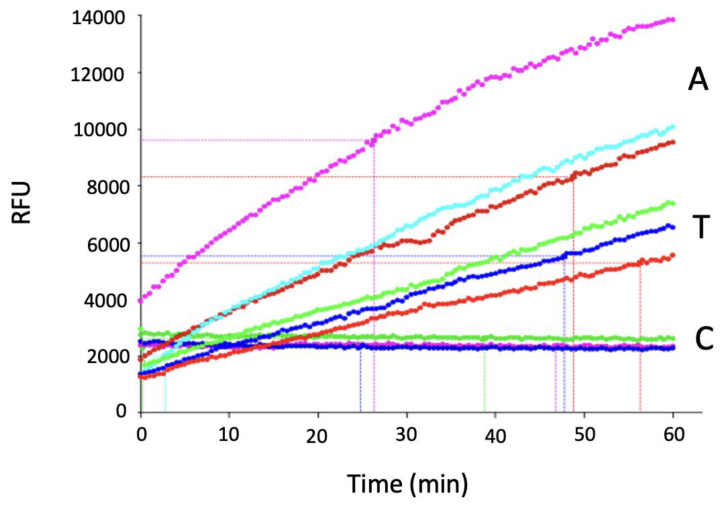
Activity of MT5-MMP toward fTHP-15. Assay was performed in triplicate, where A = activation by 4-aminophenylmercuric acetate (APMA) (light purple, light blue, and dark red lines), T = activation by trypsin (light green, blue, and red lines), and C = control (substrate alone) (green, purple, and dark blue lines). The full sequence of fTHP-15 is (Gly-Pro-Hyp)_5_-Gly-Pro-Lys(Mca)-Gly-Pro-Gln-Gly~Leu-Arg-Gly-Gln-Lys(Dnp)-Gly-Val-Arg-(Gly-Pro-Hyp)_5_-NH_2_. Methods for monitoring MMP activity towards fTHP-15 have been previously described [40]. RFUs = relative fluorescence units.

While MT5-MMP was reported to not cleave type I collagen [29], we observed that MT5-MMP could cleave fluorogenic triple-helical peptide 15 (fTHP-15), whose sequence is derived from types I-III collagen (Figure 2) [40]. Interestingly, fTHP-15 is cleaved by MT5-MMP at the Gly~Leu bond, which is the same bond cleaved by MT5-MMP in KISS-1 and metastin (Gly^118^~Leu^119^) [25].

## 3. MT5-MMP Inhibition

There are only a few inhibitors described for MT5-MMP. MT5-MMP is inhibited by the broad-spectrum MMP inhibitors GM6001 [17,33] and BB94 [29], as well as the chelator ethylenediaminetetraacetic acid (EDTA) [29]. Consistent with the general behavior of MT-MMPs, MT5-MMP is inhibited by TIMP-2, but not TIMP-1 [1,29].

MT1-MMP fragment antigen-binding (Fab) antibodies have been tested against MT5-MMP [41]. Six inhibited MT5-MMP in the 54–74% range at 100 nM Fab concentration [41].

**Figure 3:**
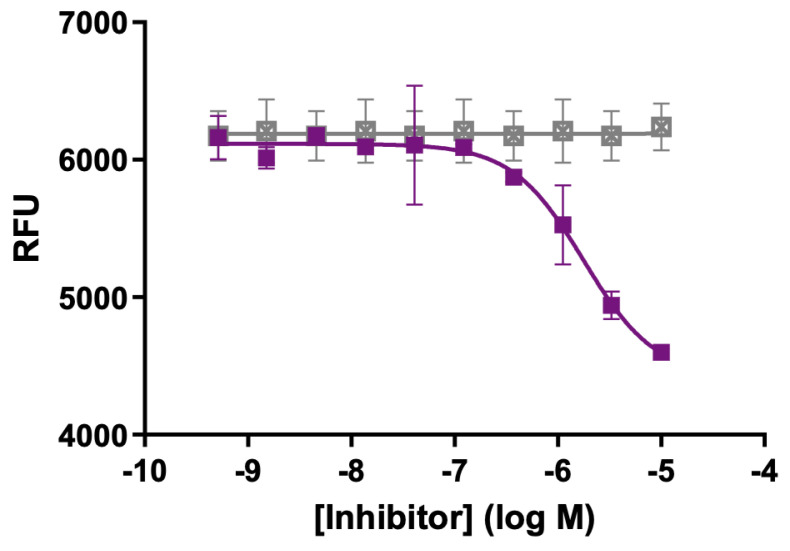
Inhibition of MT5-MMP by α1 (I-III)GlyΨ{PO_2_H-CH_2_}Leu THPI. No inhibitor (vehicle) is represented by closed gray squares, while application of inhibitor is represented by closed purple squares. The full sequence of α1 (I-III)GlyΨ{PO_2_H-CH_2_}Leu THPI is C_6_-Gly-Pro-Flp-(Gly-Pro-Hyp)_4_-Gly-Pro-Gln-GlyΨ{PO_2_H-CH_2_}(*R*,*S*)Leu-Ala-Gly-Gln-Arg-Gly-Ile-Arg-(Gly-Pro-Hyp)_4_-Gly-Pro-Flp-NH_2_. Methods for analyzing inhibition have been previously described [43,44]. RFUs = relative fluorescence units.

Designing selective inhibitors for MT5-MMP is especially challenging due to the common features of MMP active sites [42]. Based on its hydrolysis of a triple-helical substrate (Figure 2), we examined the potential inhibition of MT5-MMP by our previously described triple-helical peptide transition state analog inhibitor α1 (I-III)GlyΨ{PO_2_H-CH_2_}Leu THPI [43,44]. We found that MT5-MMP was inhibited by the THPI with an IC_50_ value of 1.8 μM (Figure 3). This result opens the possibility of selective MT5-MMP inhibition by combining transition-state analogs and MT5-MMP substrate sequences.

## 4. MT5-MMP Roles in Cancer

Members of the MMP family have long been established as contributors to cancer initiation, growth, and metastasis [45,46]. Amongst the MT-MMPs, MT1-MMP has been deemed an essential contributor to tumor invasion [47,48,49]. Thus, several studies have examined a potential role for MT5-MMP in cancer. MT5-MMP expression has been detected in breast cancer cell lines [50] and breast cancer tissue [51], with mRNA expression increased in tumor tissue compared to normal breast tissue [52]. Its expression correlates with tumor grade [52]. Roughly 20% of breast cancers lack functional repressor element 1 silencing transcription factor (REST), and these tumors are more aggressive, resulting in poorer prognoses [53,54]. REST directly regulates *MT5-MMP* expression, and the knockdown of REST leads to the upregulation of *MT5-MMP* (~45-fold) [54]. REST binds to the RE1 site in the first intron of *MT5-MMP* [54]. These studies focused on MT5-MMP expression but did not explore a mechanistic role for the enzyme in breast cancer.

Extracellular matrix stiffness occurs in the tumor microenvironment [55,56] (Paszek 2005 & Levental 2009). This stiffness can modulate tumor behaviors, including promoting invasion and proliferation. It has been proposed that binding to rigid substrates results in increased expression of MT5-MMP and slowing of the progress of breast, lung, and renal cancers [57]. Increased substrate stiffness activates the Yes-associated protein (YAP)-TEA domain (TEAD), which then promotes the expression of the enzyme via a TEAD recognition sequence upstream of the transcription start site [57]. This may explain why some studies show that higher MT5-MMP expression correlates with better survival [57].

The upregulation of MT5-MMP in non-small-cell lung cancer (NSCLC) is associated with worse progression-free survival [58]. The transcription repressor Capicua (CIC), when inactivated, results in ETV4 upregulating MT5-MMP [58]. MT5-MMP was localized to the leading edge of primary NSCLC tumors [58]. In mouse models MT5-MMP was found to promote tumor cell circulatory extravasation and stable lung colonization, as well as metastasis [58]. MT5-MMP also promotes the invasion of ovarian cancer cells [59]. As with the breast cancer studies described above, the NSCLC and ovarian cancer studies did not investigate a mechanism for MT5-MMP action.

MT5-MMP was overexpressed in gastric cancer compared with peritumoral normal tissue [60] and with chronic superficial gastritis [61], where it was suggested as a prognostic molecular marker [61]. In similar fashion to NSCLC, the inactivation of CIC in gastric cancer results in ETV4 upregulating MT5-MMP [58]. The gastric cancer studies examined MT5-MMP expression only.

In brain tumors, including astrocytomas and glioblastomas, MT5-MMP expression is high [1,13], though its functional role remains unclear [62]. An evaluation of epidermal growth factor receptor (EGFR) signaling via EGF stimulation of glioma cell lines resulted in only a slight increase (1.3- to 1.6-fold) in MT5-MMP mRNA levels [63].

Overall, correlations between increased MT5-MMP expression and cancer progression have been documented, but no specific role for MT5-MMP has been identified. Thus, at present MT5-MMP could serve as a cancer biomarker. It appears that further substrate identification will be needed in order to evaluate MT5-MMP activity in cancer invasion and metastasis.

## 5. MT5-MMP Roles in Alzheimer’s Disease

**Figure 4:**
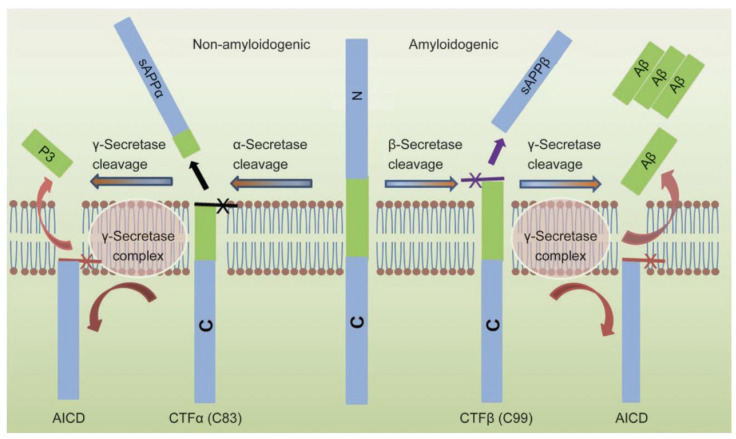
Processing of amyloid precursor protein (center) by β-secretase (right pathway) to produce sAPPβ and CTFβ/C99 or by α-secretase (left pathway) to produce sAPPα and CTFα/C83 [65]. Subsequent processing by γ-secretase produces Aβ (right pathway) or P3 (left pathway). Figure reproduced from [65] by permission of Springer Nature.

In Alzheimer’s disease (AD), the extracellular accumulation of abnormally folded amyloid beta (Aβ) peptides forms amyloid plaques. The Ab peptides are generated in a two-stage process, where APP is first cleaved by β-site APP cleaving enzyme 1 (BACE1) to produce sAPPβ and CTFβ/C99, followed by γ-secretase cleavage to produce Aβ42 and the amyloid precursor protein intracellular domain (AICD) (Figure 4) [64,65,66]. Alternatively, the cleavage of APP in the middle of the Aβ domain by α-secretase (mediated by a disintegrin and metalloprotease 10/17 (ADAM10 and ADAM17)) precludes Aβ generation (Figure 4) [64,65,66].

**Figure 5:**
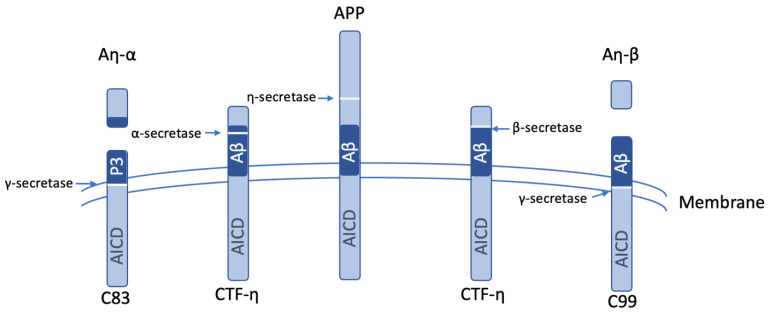
Processing of APP by η-secretase (MT5-MMP) to produce CTF-η and sAPPη/sAPP95. Subsequent processing by α-secretase produces Aη-α and C83, while subsequent processing by β-secretase produces Aη-β and C99.

MT5-MMP may have opposing effects in AD versus normal physiology [27]. The enzyme is found in post-mortem AD brains around senile plaques in dystrophic neurites, and its expression is pro-amyloidogenic [32,66,67]. PCSK6 exacerbates AD pathogenesis by promoting MT5-MMP maturation [21]. Elevated enzyme protein levels are found in the 5xFAD mouse model of AD [68]. Identified as the η-secretase involved in APP processing, MT5-MMP cleaves at residues 504–505 (Val-Leu-Ala-Asn^504^-Met^505^-Ile-Ser-Glu-Pro-Arg) (APP695 numbering) [32], contributing directly to AD pathology [32,67]. This cleavage generates a *C*-terminal membrane-bound APP fragment, CTF-η [32,67], which accumulates in distrophic neurites close to amyloid plaques [42,66,69,70]. CTF-η is localized in Golgi, endosomes, and extracellular vesicles and contributes to Aβ production [69]. Processing of CTF-η by ADAM10/17 and BACE1 releases Aη-α (108 residues) and Aη-β (92 residues), respectively (Figure 5) [32,42,69,70], which are synaptotoxic [32,66,71]. Among AD risk loci is *ADAM17* [72]. The processing of APP by η-secretase and the presence of Aη peptides have been observed in patient cerebrospinal fluid (CSF) [32,71]. Aη-β has been found to impair hippocampal long-term potentiation and inhibit neuronal activity [71], which is a crucial process for learning and memory.

MT5-MMP η-secretase activity can be considered pro-amyloidogenic because CTF-η can be processed by β-secretase and γ-secretase to yield Aβ [69]. A non-amyloidogenic route is also possible following MT5-MMP action on APP as CTF-η can be degraded by proteasomal and autophagic pathways [69]. Soluble sAPP fragment of 95 kDa (sAPP95, sAPPη), another product of η-secretase/MT5-MMP activity, binds GABA*_B_*R1a and impedes presynaptic release [66,67,73].

When β-secretase activity is pharmacologically or genetically inhibited, Aη peptide levels are increased [32]. We found that a β-secretase inhibitor increased the activity of MT5-MMP [24]. Overall, inhibiting BACE1 may result in the accumulation of alternative APP fragments such as Aη-α [64].

Reduced levels of Aη-α were observed in the brains of MT5-MMP knockout mice [32]. Crossing MT5-MMP-deficient mice with the 5xFAD AD mouse model produced bigenic mice that had reduced Aβ plaque deposition and reduced soluble Aβ and soluble APP *C*-terminal fragments (CTFs) within the brain and improved performance on a behavioral learning task [67,68,74]. These effects were maintained even after 16 months. It was proposed that MT5-MMP may influence the intracellular trafficking of APP and/or its targeting to subcellular compartments where the C-terminal fragment of APP (C99) is degraded, thus accounting for the lower Aβ40 and Aβ42 levels when MT5-MMP was absent. The levels of IL-1β decreased by 30% in the bigenic mice, which is indicative of a dampened inflammatory response due to the decreased Aβ burden in the brain [67].

MT5-MMP knockout in 5xFAD mice prevented dysfunctions of the frontal cortex (including learning and memory deficits) observed in 5xFAD mice [74]. 5xFAD/MT5-MMP^−/−^ mice had reduced Aβ assembles (soluble, oligomeric, and fibrillary) and C99 compared with 5xFAD mice [74]. More specifically, soluble Aβ38 was reduced 83%, Aβ40 84%, and Aβ42 90% [74]. Significant decreases in sAPPα and sAPPβ were also observed in 5xFAD/MT5-MMP^−/−^ mice compared with 5xFAD mice [74]. Astrocyte activity and tumor necrosis factor alpha (TNF-α) levels were also reduced [74]. MT5-MMP localized to early endosomes and increased the content of APP and Aβ40 [74]. It is interesting to note that MT5-MMP deficiency had no impact on microglial reactivity in the frontal cortex in contrast to the hippocampus [67,74].

An examination of neuronal cultures revealed that the absence of MT5-MMP impaired the IL-1β-mediated induction in inflammatory genes in cells from 5xFAD mice compared with cells from 5xFAD mice where MT5-MMP was not deleted [27]. MT5-MMP can activate proinflammatory pathways [75]. Its deficiency decreased C83 and C99 levels (which are derived from CTF-η; see Figure 5); these fragments are cleared in the absence of MT5-MMP [27].

The deletion of the MT5-MMP *C*-terminal domain reduced its ability to process APP and release sAPP95 [76]. This included the deletion of the intracellular domain, transmembrane domain + intracellular domain, or hemopexin-like domain [76]. The *C*-terminal domain of MT5-MMP directly interacted with CTFβ/C99 [76], and thus when this domain was deleted CTFβ/C99 could be degraded by the proteasome, preventing Aβ accumulation [76]. MT5-MMP may regulate APP cellular (endo-lysosomal) trafficking as both active and inactive enzymes increased extracellular levels of Aβ40 [76]. MT5-MMP increased the content of APP/Aβ in early endosomes [76]. Its non-catalytic domains contributed to the MT5-MMP-catalyzed production of sAPP95 [76]. It was proposed that an APP/Mint3/MT5-MMP trimolecular complex could promote APP/C99 trafficking to endosomes [76]. Similar *C*-terminal interactions were not found in MT1-MMP [76], and thus this trafficking may be unique for MT5-MMP.

It has been hypothesized that the role of MT5-MMP in AD is multifaceted [75]. MT5-MMP directly acts on APP, but it could also traffic APP to endosomal/lysosomal compartments and enhance proinflammatory responses induced by IL-1β or TNF-α due to N-cadherin processing [75].

The endosome has been reported as the main site for the MT5-MMP processing of APP in transient transfection studies [69,74]. MT5-MMP could facilitate the β-secretase processing of APP in endosomes [66]. In addition to endosomes [66] the Golgi has recently re-emerged as a major location for APP processing and Aβ production [77]. MT5-MMP can locate to the trans-Golgi network to further recycle to the membrane even though it lacks a di-leucine motif in its *C*-terminal tail [23]. We stably co-expressed APP751 and MT5-MMP-GFP in CHO cells and found that the enzyme localized in cytosolic subcellular granules [24]. Consistent with our observation that MT5-MMP is found in the Golgi apparatus, CTF-η has been found to colocalize with TGN-46, a marker for the Golgi apparatus and trans-Golgi network [69]. CTF-η fragments can ultimately be transported via exosomes [69], while Aβ peptides can be transported outside the cell by HSP47 [78].

The *C*-terminus of MT5-MMP was utilized as a bait to screen the human brain cDNA library [24]. MT5-MMP was found to directly bind to transmembrane protein 199 (TMEM199), NEDD4-binding protein 2-like 1 (N4BP2L1), thioredoxin related transmembrane protein 4 isoform X3 (TMX3), bridging integrator 1 (BIN1), RUFY4, high temperature requirement protein A1 (HTRA1), and endosome/lysosome-associated apoptosis and autophagy regulator (also called estrogen induced gene 121; transmembrane protein KIAA1324) (EIG121) [24]. The binding of N4BP2L1, TMX3, BIN1, TMEM199, or EIG121 to MT5-MMP increased Aη-α and Aη-β production [24]. The association of several of these binding partners can be linked to MT5-MMP localization to the Golgi apparatus, based on the interaction between lysosomes and the Golgi apparatus [79]. The transmembrane nature of MT5-MMP suggests the incorporation of the enzyme into endosomes and lysosomes during vacuole formation. The protein encoded by TMEM199 has been observed to localize to the endoplasmic reticulum (ER)-Golgi intermediate compartment (ERGIC) and coat protein complex I (COPI) [80]. As the loss of *TMEM199* results in the over-acidification of the endo-lysosomal compartments [81], the processing of APP by the MT5-MMP•TMEM199 complex may be related to the accumulation of CTFβ and Aβ in poorly acidified autolysosomes [82].

EIG121 is a transmembrane protein localized in the endo-lysosomal compartments [83], and thus MT5-MMP’s association with EIG121 may occur in the endosomes and lysosomes. MT5-MMP has been shown to be associated with early endosomes [76], while BIN1 contributes to early-endosome size deregulation, which is an early pathophysiological hallmark of AD pathology [84]. Thus, the association between BIN1 and MT5-MMP may occur in early endosomes. *BIN1* has been identified as an AD susceptibility gene [84].

RUFY4 promotes the coupling of endo-lysosomes to dynein–dynactin for retrograde transport along microtubules [85], and thus the association of MT5-MMP with RUFY4 could occur in the endosomes and lysosomes. HTRA1 is a secreted serine protease that degrades various fragments of the amyloid precursor protein and colocalizes with β-amyloid deposits in human brain samples [86]. One cannot speculate where HTRA1 or TMX3 are associated with MT5-MMP due to the relative lack of information pertaining to their function [24]. TMX3 (PDIA13) is an endoplasmic reticulum oxidoreductase/disulfide isomerase [87,88], while N4BP2L1 has been identified as a highly significant differentially expressed gene in AD [89]. It is not readily apparent how most of the MT5-MMP binding partners enhance proteolytic activity.

TIMP-3 is increased in AD patient brains and APP transgenic mice [90]. TIMP-3 inhibits ADAM10 and ADAM17 and thus routes APP processing away from α-secretase and instead enhances β-secretase activity and endocytosis [90]. This would deter the production of Aη-α. TIMP-3 plasma and cerebrospinal fluid levels are lower in AD patients compared with non-AD patients [91]. It has been suggested that TIMP-3 could aggregate in the brain in AD [91].

## 6. MT5-MMP Roles in Other Pathologies

MT5-MMP is elevated 2 and 7 days after traumatic brain injury (TBI), while N-cadherin protein decreases [92]. The two proteins localize within reactive astrocytes, which produce MT5-MMP during reactive synaptogenesis [92]. As N-cadherin links and stabilizes presynaptic terminals with postsynaptic structures, this remodeling activity may be crucial in post-TBI synapse reorganization [92]. Given the increase in proMMP-2 in TBI, it is possible that MT5-MMP may activate MMP-2, resulting in matrix turnover [92].

MT5-MMP is expressed in the epithelial tissue in the normal cornea [93]. *P. aeruginosa* infection induces its expression in the substantia propria [93]. The levels of enzyme were found to increase 7 days after infection, possibly due to the infiltration of macrophages [93]. While it was noted that MT5-MMP hydrolyzes a number of extracellular matrix components (such as fibronectin and proteoglycans) and hydrolysis of these components may contribute to cornea damage, no specific mechanism was explored.

Kidney MT5-MMP expression was localized to the epithelial cells of the proximal and distal convoluted tubules in the cortex, the collecting duct, and the loop of Henle in the medulla [13]. MT5-MMP mRNA and protein are increased in the diabetic kidney [13]. MMP-2 activity was also increased in the diabetic kidney [13]. Tubular epithelial cells were found to be associated with tubular atrophy [13]. It was proposed that during diabetic nephropathy, MT5-MMP and MMP-2 remodeled the basement membrane, resulting in tubular epithelial cell detachment, apoptosis of epithelial cells, and tubular atrophy.

MT5-MMP expression is found in normal human endometria and is elevated in endometriosis [26]. More specifically, *MT5-MMP* showed an 8.4-fold higher expression in endometriotic lesions in comparison with endometrium [26]. MT5-MMP protein was detected in luminal epithelial cells [26]. In the evaluation of the roles of MMPs in the premature rupture of membranes (PROM) during labor and delivery, MT5-MMP was ultimately not considered a contributor [94].

## 7. Conclusions

MT5-MMP upregulation has been observed in a variety of disease states, but the majority of studies are correlative without proposed mechanisms of action. The most detailed studies of MT5-MMP define the role of the enzyme in AD. In the case of AD, the presence of the η-secretase and β-secretase pathways may be the reason for failed clinical trials when targeting Aβ reduction only [95]. Approaches to decrease the activity of BACE1 and limit the activity of MT5-MMP will shift the pathway from pro-amyloidogenic processing to the non-amyloidogenic one. The development of selective MT5-MMP inhibitors could also be utilized to better define the enzyme’s role in a variety of cancers. An additional approach for regulating MT5-MMP could be targeting activators of the enzyme, such as furin or PCSK6. As noted, MT5-MMP knockout mice are viable and have no overt abnormalities, suggesting that MT5-MMP could be a therapeutic target in AD and cancer [18,42,75]. Overall, the substrate profile of MT5-MMP has not been well defined, and selective inhibitors of MT5-MMP have not been described. These advances will be needed for further consideration of MT5-MMP as a therapeutic target.

## Data Availability

Not applicable.
